# 

**Published:** 1858-07

**Authors:** 


### CONTENTS.

ORIGINAL COMMUNICATIONS— PAGE.

Successful Case of Resection of the Knee Joint, with Remarks, and a Table of most of the Resections of the Knee performed to the present time. By Daniel Brainard, M. D. ........ 293
Placenta Prævia. By J. P. Terrell, M. D. ........ 297
Case of Pseudo-Membranous Laryngitis, or True Croup. By J. Drake Harper, M.D. ........ 300
Measles a Second Time in the Same Patients. By D. W. Young, M.D. 304
Medical Education. By J. H. Brown, M.D. ........ 306
Case of Peritonæal Inflammation. By W. C. Haines, M.D. ........ 308
Inhalation of Nitrate of Silver. By W. H. Studly, M.D. ........ 309
What are the Causes which have brought about a Change in the General Treatment of our Diseases, from the Depletive to a Tonic and Stimulating? By J. M. Hinkle, M.D. ........ 311
Translations by E. L. Holmes, M.D.—
Upon the Treatment of Tabes Dorsalis, by means of Uninterrupted Galvanic Currents. By R. Remak ........ 318
Rheumatism ........ 322
Spontaneous Mortification, following Obstruction of the Arteries ...... 324

PROCEEDINGS OF MEDICAL SOCIETIES—

Semi-Annual Meeting of the Esculapian Society, held in Marshall, Ill.... 326
The Rock Island County Medical Society ........ 329

BOOK AND PAMPHLET NOTICES—

Review of Tully's Materia Medica. By Ira Hatch, M.D. ........ 330
Elements of Inorganic Chemistry ........ 334
Plates Illustrative of Wilson on Diseases of the Skin ........ 384
New Journals ........ 336

EDITORIAL—

A Plain Talk with the Readers of the Journal ........ 337
Eleventh Annual Meeting of the American Medical Association ........ 340
Report on the Sanitary Condition of Chicago. By N. S. Davis ........ 342

